# Decitabine facilitates thrombopoiesis independent of the thrombopoietin receptor in zebrafish

**DOI:** 10.1097/BS9.0000000000000216

**Published:** 2025-02-11

**Authors:** Yudong Chen, Liangliang Wu, Lian Yang, Jiajun Chen, Xiaofan Gu, Yiyue Zhang, Qing Lin

**Affiliations:** a The Innovation Centre of Ministry of Education for Development and Diseases, School of Medicine, South China University of Technology, Guangzhou 510006, China; b Department of Hematology, Guangzhou First People’s Hospital, South China University of Technology, Guangzhou, Guangdong, China; c Department of Hematology, the Sixth Affiliated Hospital, School of Medicine, South China University of Technology, Foshan, Guangdong China

## 1. INTRODUCTION

Thrombocytopenia, including immune thrombocytopenia (ITP), aplastic anemia, myelodysplastic syndrome, hereditary thrombocytopenia, and isolated thrombocytopenia after hematopoietic stem cell transplantation (HSCT), is defined as a platelet count of <150 × 10^9^/L and may lead to spontaneous bleeding, prolonged bleeding, and menorrhagia.^1–3^ The induction of thrombopoiesis and elevation of platelet count using thrombopoietin (THPO) receptor agonists (THPO-RAs) are critical treatments for thrombocytopenia.^4^ However, many patients with thrombocytopenia do not respond to THPO-RAs and require additional therapies.^5^ Moreover, THPO-RA treatment has been proven ineffective in patients with hereditary thrombocytopenia caused by mutations in the human myeloproliferative leukemia (MPL) protein gene. Novel treatment modalities, beyond transfusion and HSCT, are needed for these forms of thrombocytopenia.^6,7^

In our previous study, we demonstrated that decitabine effectively alleviated thrombocytopenia in *gp9*^*SMU15*^ mutant zebrafish.^8^ Previous studies have shown that low-dose decitabine administration significantly increases platelet count by promoting the maturation of polyploid megakaryocytes, enhancing thrombopoiesis, and facilitating their release in vivo and in vitro.^9,10^ Moreover, prospective multicenter studies have shown that low-dose decitabine is an effective treatment for patients with refractory ITP and refractory prolonged isolated thrombocytopenia after allogeneic HSCT.^2,7^ However, megakaryocytopoiesis and thrombopoiesis primarily depend on THPO stimulation of the MPL.^11,12^ Whether the effect of decitabine on platelet count elevation is dependent on MPL signaling remains unclear.

Previously, we established zebrafish models of congenital thrombocytopenia, *Tg(mpl:eGFP)smu4;mpl*^*smu3*^. This zebrafish model,^13^ carrying an *mpl* mutation, replicates human congenital amegakaryocytic thrombocytopenia (CAMT). Thrombocytes were uniquely identified based on their green fluorescent protein (GFP) expression. Therefore, we investigated the effect of decitabine on *mpl*-mutant zebrafish to determine whether decitabine could increase thrombocyte counts independently of *Mpl* signaling. We found that decitabine effectively increased thrombocyte count.

Moreover, decitabine facilitated thrombopoiesis by promoting the proliferation of thrombocyte precursors (*cd41:eGFP*^*+*^ cells) in *Tg(cd41:eGFP);mpl*^*smu3*^ zebrafish, indicating that the effect of decitabine on thrombopoiesis is independent of *Mpl* signaling. Similarly, elevated thrombocyte counts were observed in a *thpo* expression-deficient zebrafish model (*thpo*^*szy6*^).^14^ These results suggest the potential application of decitabine in the treatment of congenital thrombocytopenia caused by *MPL* mutations, as well as in thrombocytopenia that does not respond to THPO-RA treatment.

## 2. MATERIALS AND METHODS

### 2.1. Maintenance of fish strains

Zebrafish were maintained under standard conditions (28.5°C, 14-hour light/10-hour dark). The following zebrafish strains were used: AB (wild type), *Tg(mpl:eGFP)smu4*, *Tg(mpl:eGFP)smu4*;*mpl*^*smu3*^, *Tg(cd41:eGFP), Tg(cd41:eGFP);mpl*^*smu3*^, *thpo*^*szy6*^. All protocols involving zebrafish were approved by the Institutional Animal Care and Use Committee of South China University of Technology.

### 2.2. Treatment with decitabine agents

Zebrafish embryos were incubated in egg water containing decitabine (MedChemExpress, New Jersey) starting 24-hour post-fertilization (hpf) until the desired stage.

### 2.3. Immunohistochemistry and anti-phosphorylated histone H3 immunostaining

Immunohistochemistry and anti-phosphorylated histone H3 (pH3) immunostaining were performed as previously described.^14,15^ Embryos were stained with goat anti-GFP (1:400; Abcam, Cambridge, UK) to detect GFP expression, and GFP staining was visualized using Alexa Fluor donkey anti-goat-488 (1:400; Invitrogen, Massachusetts). For pH3 expression, embryos were stained with rabbit anti-phosphohistone H3 antibody (Santa Cruz Biotechnology, Texas), followed by Alexa Fluor 594-conjugated anti-rabbit secondary antibody (1:400; Invitrogen).

### 2.4. Whole-mount in situ hybridization (WISH)

The cRNAs of probes (β*e1, mpl, rag1, c-myb,* and *runx1*) were transcribed in vitro using T3 or T7 polymerase (Thermo Fisher Scientific, Massachusetts) with a digoxigenin-labeled NTP mix (Roche, Basel, Switzerland). WISH was performed as previously described.^16^

### 2.5. Sudan Black (SB) staining

Fixed embryos were incubated in SB solution (Sigma-Aldrich, Massachusetts) for 30 minutes and counted as previously described.^17^

### 2.6. Bromodeoxyuridine (BrdU) labeling

BrdU labeling was performed as previously described.^14^ Embryos at 3 days post-fertilization (dpf) were incubated in egg water containing 10 mM BrdU for 6 hours at 28.5°C. The embryos were then treated with 2 N HCl for 1 hour at 25°C and incubated with anti-GFP goat serum (1:400; Invitrogen), followed by Alexa Fluor 488-conjugated donkey anti-goat secondary antibody (1:400; Invitrogen), mouse anti-BrdU primary antibody (1:50; Roche), and Alexa Fluor 555-conjugated goat anti-mouse secondary antibody (1:400; Invitrogen). Images were captured using a Zeiss LSM800 confocal microscope.

### 2.7. Statistical analysis

Categorical variables were analyzed using the Fisher exact test. Continuous variables were analyzed using the Student *t* test. Data are expressed as means ± standard deviation (SDs). Statistical analyses were performed using GraphPad Prism software (version 7.0). A two-tailed *P* < 0.05 was considered statistically significant.

## 3. RESULTS

### 3.1. Decitabine increased thrombocyte counts by promoting the proliferation of thrombocyte precursors independent of the THPO/MPL pathway

**Figure 1.**
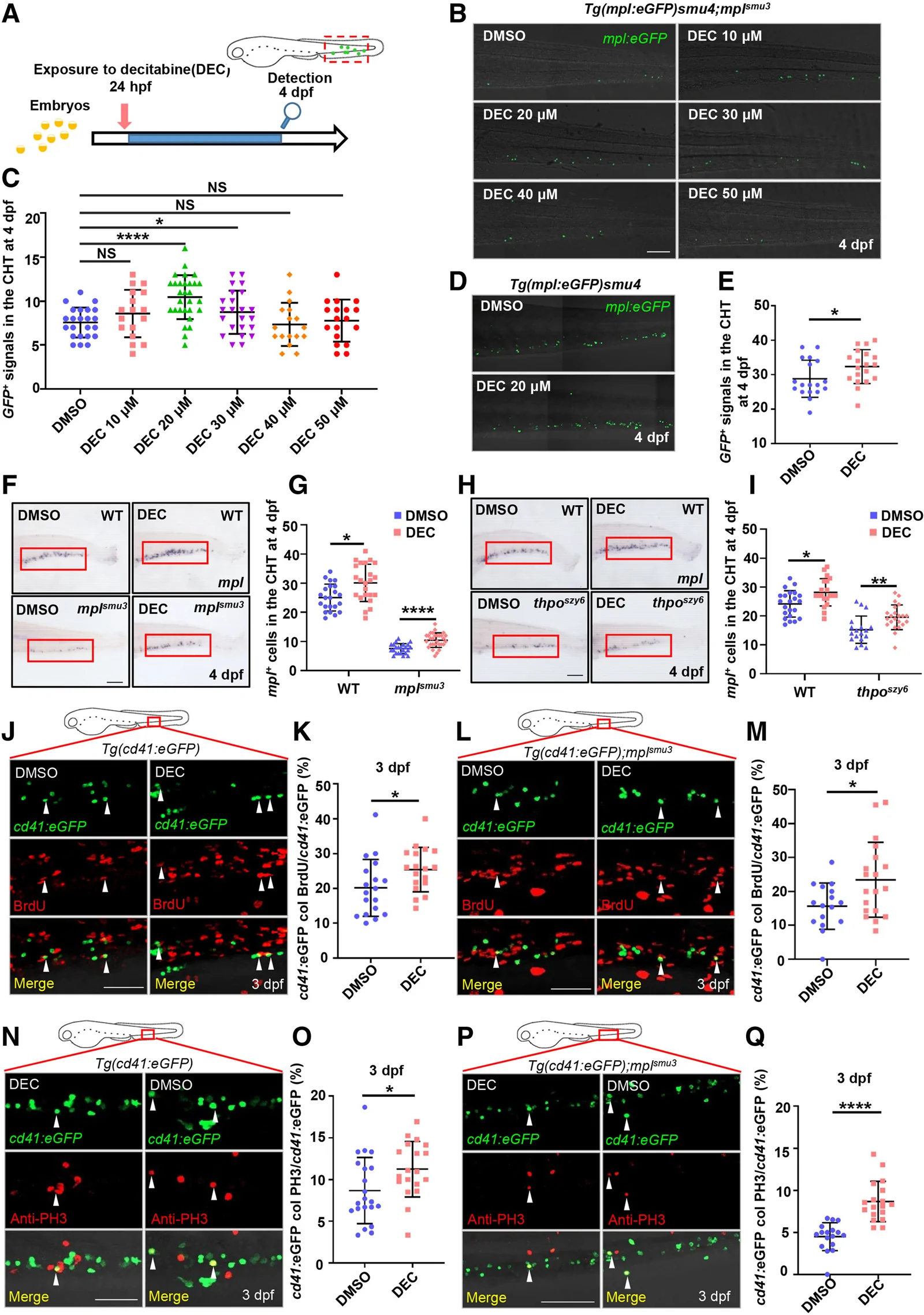
Decitabine treatment increases thrombocyte counts in zebrafish. Time course of drug treatment. (A) Embryos were exposed to decitabine from 24 hpf to 4 dpf, and the *mpl:eGFP*^*+*^ cells in the CHT were counted at 4 dpf. (B) Immunofluorescence staining of *eGFP* in the CHT of control and decitabine-treated *Tg(mpl:eGFP);mpl*^*smu3*^ groups at 4 dpf. Scale bars: 100 μm. (C) Statistical analysis of *mpl:eGFP*^*+*^ cells in the CHT of groups treated with different concentrations of decitabine at 4 dpf. Student *t* test, n > 15, mean ± SD. *P < 0.05; ****P < 0.0001. (D) Immunofluorescence staining of *eGFP* in the CHT of control and decitabine-treated *Tg(mpl:eGFP*) groups at 4 dpf. Scale bars: 100 μm. (E) Statistical analysis of *mpl:eGFP*^*+*^ cells in the CHT of groups treated with 20 μM decitabine at 4 dpf. Student *t* test, n > 15, mean ± SD. *P < 0.05. (F) WISH for *mpl* expression in the control and decitabine-treated groups of wild-type siblings and *mpl*^*smu3*^ at 4 dpf. Scale bars: 100 μm. (G) Statistical analysis of *mpl*-positive CHT cells by WISH in the control and decitabine treatment groups of wild-type siblings and *mpl*^*smu3*^ at 4 dpf. Student *t* test, n > 17, mean ± SD. *P < 0.05; ****P < 0.0001. (H) WISH for *mpl* expression in the control and decitabine-treated groups of wild-type siblings and *thpo*^*szy6*^ at 4 dpf. Scale bars: 100 μm. (I) Statistical analysis of *mpl*-positive CHT cells by WISH in the control and decitabine treatment groups of wild-type siblings and *thpo*^*szy6*^ at 4 dpf. Student *t* test, n > 15, mean ± SD. *P < 0.05; **P < 0.01. (J) Representative image of BrdU incorporation by CHT *cd41:eGFP+* cells in 3dpf *Tg(cd41:eGFP*) zebrafish by double antibody staining of BrdU (red signals) and GFP (green signals) in control and decitabine-treated groups. White triangular arrowheads indicate yellow-colored merged signals representing the BrdU^+^*cd41*^*+*^ cells. Scale bars: 50 μm. (K) Statistical analysis showing the percentages of CHT-localized *cd41*^*+*^*:eGFP* cells that incorporated BrdU. Student *t* test, n > 15, mean ± SD. *P < 0.05. (L) Representative image of BrdU incorporation by CHT *cd41:eGFP+* cells in 3dpf *Tg(cd41:eGFP);mpl*^*smu3*^ mutant zebrafish, shown by double antibody staining of BrdU (red signals) and GFP (green signals) in control and decitabine-treated groups. White triangular arrowheads indicate the yellow color merged signals representing the BrdU^+^*cd41*^*+*^ cells. Scale bars: 50 μm. (M) Statistical analysis showing the percentages of CHT-localized *cd41*^*+*^*:eGFP* cells that incorporated BrdU. Student *t* test, n > 15, mean ± SD. *P < 0.05. (N) Representative image of anti-pH3 immunostaining in CHT *cd41:eGFP+* cells in 3dpf *Tg(cd41:eGFP*) zebrafish, shown by double antibody staining of pH3 (red signals) and GFP (green signals) in control and decitabine-treated groups. White triangular arrowheads indicate the yellow-colored merged signals representing the pH3^+^*cd41*^*+*^ cells. Scale bars: 50 μm. (O) Statistical analysis showing the percentages of CHT-localized *cd41*^*+*^*:eGFP* cells that incorporated pH3. Student *t* test, n > 15, mean ± SD. *P < 0.05. (P) Representative image of anti-pH3 immunostaining in CHT *cd41:eGFP+* cells in 3dpf *Tg(cd41:eGFP);mpl*^*smu3*^ mutant zebrafish, shown by double antibody staining of pH3 (red signals) and GFP (green signals) in control and decitabine-treated groups. White triangular arrowheads indicate the yellow-colored merged signals representing the pH3^+^*cd41*^*+*^ cells. Scale bars: 100 μm. (Q) Statistical analysis showing the percentages of CHT-localized *cd41*^*+*^*:eGFP* cells that incorporated pH3. Student *t* test, n > 15, mean ± SD. ****P < 0.0001. BrdU = bromodeoxyuridine, CHT = caudal hematopoietic tissue, GFP = green fluorescent protein, NS = not significant, SD = standard deviation, WISH = whole-mount in situ hybridization.

To identify the optimal concentration of decitabine for increasing thrombocyte counts independent of *Mpl* signaling, 24 hpf *Tg(mpl:eGFP)smu4*;*mpl*^*smu3*^ embryos, characterized by *mpl*-deficiency and congenital thrombocytopenia, with thrombocytes specifically marked by GFP expression, were treated with different concentrations (10–50 μM) of decitabine for 3 days (**Fig. 1A**). As shown in Figure 1B and C, treatment with 20 µM decitabine resulted in the greatest increase in the number of GFP+ cells in the caudal hematopoietic tissue (CHT) of 4 dpf *Tg(mpl:eGFP)smu4*;*mpl*^*smu3*^ zebrafish compared to other concentrations of decitabine. This finding indicated that the effect of decitabine on thrombopoiesis was dose-dependent, with 20 µM being the optimal concentration. Subsequent experiments using this concentration revealed that 20 μM decitabine increased the number of GFP+ cells in wild-type siblings (**Fig. 1D and E**). Additionally, WISH results demonstrated a significant increase in the expression of the thrombocyte marker *mpl* in the CHT of wild-type siblings and *mpl*^*smu3*^ zebrafish after treatment with 20 µM decitabine (**Fig. 1F and G**). To further confirm whether the increased thrombocyte counts observed after decitabine treatment was associated with changes in *thpo* expression, we conducted a decitabine administration experiment in *thpo*^*szy6*^ zebrafish, a thrombocytopenia model characterized by a significant deficiency in *thpo* expression.^14^ Our findings are consistent with the results observed from *mpl*^*smu3*^ zebrafish, which showed that both wild-type siblings and *thpo*^*szy6*^ zebrafish exhibited higher thrombocyte counts under the same treatment conditions (**Fig. 1H and I**). These results suggest that decitabine treatment promotes increased thrombocyte counts independent of Thpo signaling.

To understand how decitabine promotes thrombopoiesis in the treated embryos, we investigated whether thrombocyte proliferation increased. BrdU and anti-pH3 immunostaining were used to detect changes in the proportion of proliferating cells within the populations of thrombocyte precursors at 3 dpf *Tg(cd41:eGFP*) and *Tg(cd41:eGFP);mpl*^*smu3*^ mutant zebrafish. The BrdU incorporation assay revealed a significant increase in the percentage of BrdU-positive *cd41:eGFP*^*+*^ cells among the total *cd41:eGFP*^*+*^ cell population in 3 dpf *Tg(cd41:eGFP*) and *Tg(cd41:eGFP);mpl*^*smu3*^ mutant zebrafish compared to controls (**Fig. 1J–M**). Similarly, an increase of pH3 and *cd41:eGFP* double-positive cells was observed (**Fig. 1N-Q**), indicating that the proliferation of thrombocyte lineage cells was enhanced in both *Tg(cd41:eGFP*) and *mpl*^*smu3*^ mutant zebrafish. Collectively, these results indicate that the decitabine-induced increase in thrombocyte counts was achieved by promoting the proliferation of thrombocyte precursors independent of the THPO/MPL pathway.

### 3.2. Decitabine improved *mpl*^*smu3*^ thrombocyte function without affecting hematopoietic stem progenitor cells (HSPCs) or other blood cell lineages

**Figure 2.**
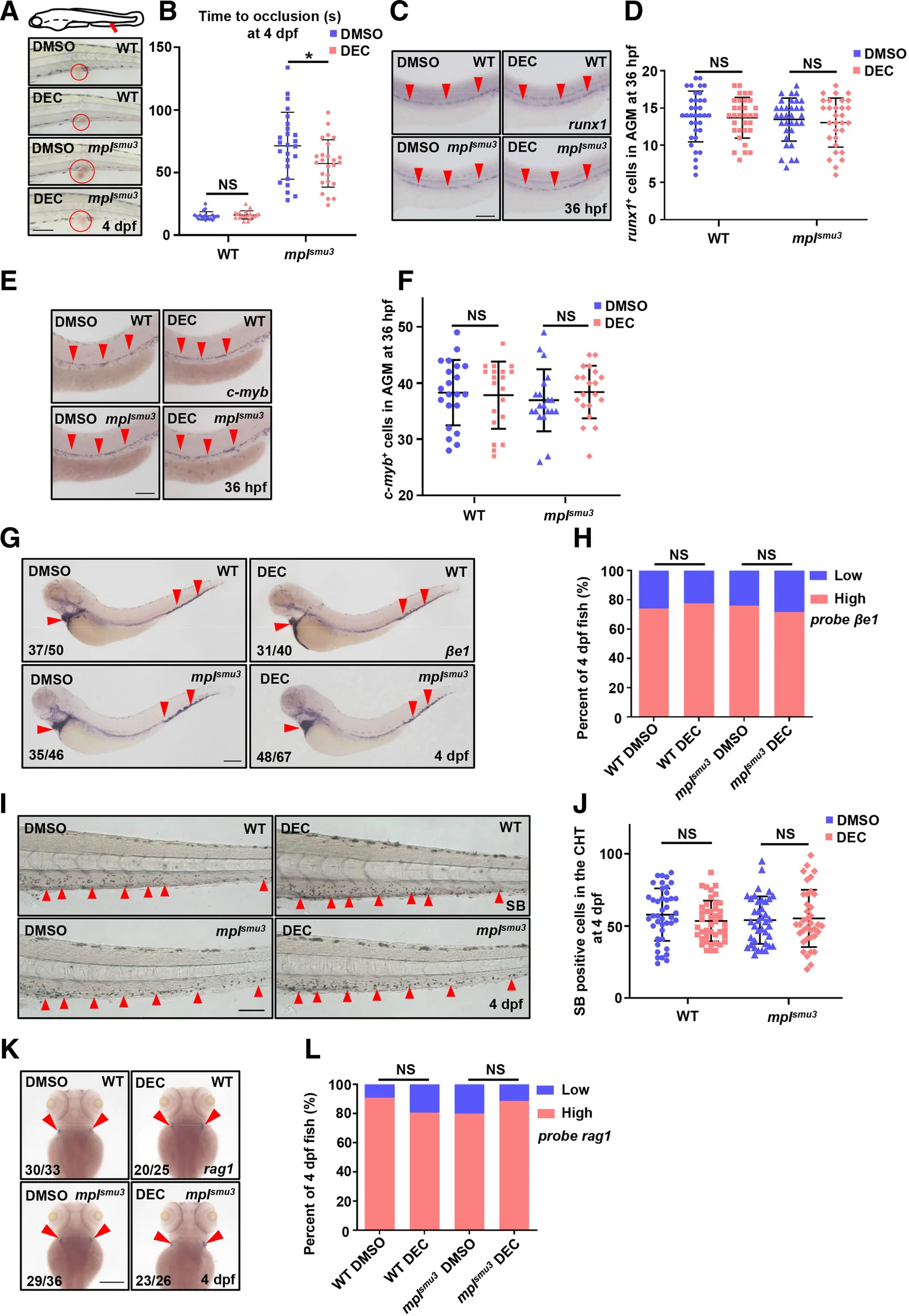
Decitabine treatment improved thrombocyte function without affecting HSPCs or other blood cell lineages. (A) Representative images showing bleeding 3 min after injury in the wild-type siblings and *mpl*^*smu3*^ mutant in control and decitabine-treated groups. The red arrow indicates the injury site, and the bleeding areas are circled in red. Scale bars: 200 μm. (B) Statistical analysis of TTO in wild-type siblings and *mpl*^*smu3*^ mutants in control and decitabine-treated groups. Student *t* test, n > 15, mean ± SD. *P < 0.05. (C) WISH for *runx1* expression in control and decitabine-treated groups of wild-type siblings and *mpl*^*smu3*^ mutants at 36 hpf. Scale bars: 100 μm. (D) Statistical analysis of *runx1*-positive cells in AGM by WISH in control and decitabine treatment groups of wild-type siblings and *mpl*^*smu3*^ mutants at 36 hpf. Student *t* test, n > 25, mean ± SD. (E) WISH for *c-myb* expression in control and decitabine-treated groups of wild-type siblings and *mpl*^*smu3*^ mutants at 36 hpf. Scale bars: 100 μm. (F) Statistical analysis of *c-myb*-positive cells in AGM by WISH in control and decitabine treatment groups of wild-type siblings and *mpl*^*smu3*^ mutants at 36 hpf. Student *t* test, n > 15, mean ± SD. (G) WISH for β*e1* expression in control and decitabine-treated groups of wild-type siblings and *mpl*^*smu3*^ mutants at 4 dpf. Scale bars: 100 μm. (H) Statistical analysis of β*e1*-positive cells in the CHT by WISH in control and decitabine treatment groups of wild-type siblings and *mpl*^*smu3*^ mutants. Fisher exact test, n ≥ 40. (I) SB staining in control and decitabine-treated groups of wild-type siblings and *mpl*^*smu3*^ mutants at 4 dpf. Scale bars: 100 μm. (J) Statistical analysis of SB-positive cells in the CHT by SB staining in control and decitabine treatment groups of wild-type siblings and *mpl*^*smu3*^ mutants. Student *t* test, n > 15, mean ± SD. (K) WISH for *rag1* expression in control and decitabine-treated groups of wild-type siblings and *mpl*^*smu3*^ mutants at 4 dpf. Scale bars: 200 μm. (L) Statistical analysis of *rag1*-positive cells by WISH in control and decitabine treatment groups of wild-type siblings and *mpl*^*smu3*^ mutants. Fisher exact test, n > 25. AGM = aorta-gonad-mesonephros, CHT = caudal hematopoietic tissue, HSPCs = hematopoietic stem progenitor cells, NS = not significant, SB = Sudan Black, SD = standard deviation, TTO = time to occlusion, WISH = whole-mount in situ hybridization.

Similar to patients with CAMT, *mpl*^*smu3*^ zebrafish exhibited defective hemostasis and an increased bleeding tendency.^13^ The efficiency of vessel clotting was evaluated to investigate the effect of decitabine on thrombocyte function. We found that decitabine did not alter the time to occlusion (TTO) in wild-type siblings. However, in *mpl*^*smu3*^ mutant zebrafish treated with decitabine, the TTO was significantly shorter than that in the untreated controls (**Fig. 2A and B**). This suggests that decitabine does not affect hemostasis in normal zebrafish but may improve it in *mpl*^*smu3*^ mutants.

HSPCs are generated from the ventral wall of the dorsal aorta at approximately 28 hpf after circulation begins and subsequently migrate to the CHT for expansion.^18,19^ To determine whether HSPCs were affected by decitabine, we analyzed *runx1* and *c-myb* expression in wild-type siblings and *mpl*^smu3^ zebrafish embryos treated with decitabine beginning at 1 dpf. The results showed no significant changes in *runx1* and *c-myb* expression in the aorta-gonad-mesonephros (AGM) region at 36 hpf (**Fig. 2C–F**). These results suggest that decitabine treatment increased thrombocyte counts without affecting HSPCs in wild-type siblings and *mpl*^smu3^ zebrafish. To further characterize the effects of decitabine treatment on hematopoiesis, erythroid, myeloid, and lymphoid cells were analyzed using WISH and SB. The results indicated no significant differences in the erythroid, myeloid, and lymphoid cell populations between groups treated with decitabine or DMSO in wild-type siblings and *mpl*^smu3^ (**Fig. 2G–L**). Thus, decitabine treatment did not affect HSPCs or the hematopoiesis of other lineages in wild-type siblings or *mpl*^smu3^ mutant zebrafish.

## 4. DISCUSSION

The interaction between THPO and MPL is essential for regulating megakaryocyte maturation and thrombocyte production. Severe thrombocytopenia of patients with CAMT and patients with *THPO* mutations provides the most convincing evidence that the THPO/MPL pathway is essential for thrombopoiesis.^6,20^ THPO-RAs have been used as a critical treatment for thrombocytopenia.^4^ However, some patients with thrombocytopenia do not respond to treatment with THPO-RAs.^5^ Available treatments for patients with CAMT include platelet transfusion and HSCT, but not THPO-RA due to MPL mutations. Drugs that promote thrombopoiesis independent of the classic THPO/MPL pathway are urgently needed for patients with thrombocytopenia who do not respond to THPO-RA or CAMT treatment.

The primary role of decitabine is its demethylating effect on gene expression regulation.^21^ Decitabine treatment promotes the differentiation of mouse bone marrow cells and the megakaryoblastic cell line L8057 into CD41a^+^ cells, thereby increasing platelet release.^10^ This effect is achieved by enhancing the expression of tumor necrosis factor-related apoptosis-inducing ligand, which plays a lineage-specific regulatory role by reducing promoter methylation status.^9^ In our study, we identified 20 μM as the optimal concentration of decitabine. Although we did not specifically assess its demethylation effects, our findings showed that decitabine increased thrombocyte numbers in both wild-type siblings and *mpl*^*smu3*^ and *thpo*^*szy6*^ zebrafish, independent of the THPO/MPL pathway. Additionally, previous work from our group demonstrated that a 20 μM concentration of decitabine significantly increased thrombocyte counts in a Bernard-Soulier syndrome model involving *gp9*^*SMU15*^ mutant zebrafish.^8^ Supporting our findings, multicenter prospective studies have shown that low-dose decitabine can effectively increase platelet counts in over half of patients with refractory ITP and in those with prolonged isolated thrombocytopenia following allogeneic HSCT.^2,7^

Moreover, we found that decitabine reduced the TTO in *mpl*^*smu3*^ mutants, suggesting its potential role in enhancing hemostasis. In addition, thrombopoiesis depends on crosstalk between megakaryocytes and bone marrow endothelial cells within the bone marrow vascular microenvironment.^22^ Decitabine treatment may also ameliorate the impairment of bone marrow endothelial progenitor cells and moderate immune dysfunction in the hematopoietic microenvironment by promoting thrombopoiesis.^23,24^ Further studies are warranted to determine the effects of decitabine treatment on endothelial cells in *mpl*^*smu3*^ mutants.

## 5. CONCLUSIONS

Our findings show that decitabine treatment increased thrombocyte numbers in *mpl*^*smu3*^ and *thpo*^*szy6*^ mutant zebrafish by promoting the proliferation of thrombocyte precursors, suggesting that decitabine enhances thrombocytopoiesis independently of the THPO/MPL pathway. Furthermore, this study indicates that decitabine treatment may be beneficial for patients with CAMT and thrombocytopenia who do not respond to THPO-RAs.

## ACKNOWLEDGMENTS

This work was supported by The National Nature Science Foundation of China (32270875 and 32370874), the Guangzhou General Science and Technology Project of Health and Family Planning (20241A011012), and the Guangzhou Municipal Science and Technology Project (2024A03J1021).

## AUTHOR CONTRIBUTIONS

Y.Z. and Q.L. designed the study. Y.C., L.W., L.Y., J.C., X.G. performed experiments. Y.C. and L.W. analyzed and organized data. Y.C. and L.W. prepared figures and drafted the manuscript. Y.Z. and Q.L. revised the manuscript.
